# Stomatal Lineage Control by Developmental Program and Environmental Cues

**DOI:** 10.3389/fpls.2021.751852

**Published:** 2021-10-11

**Authors:** Soon-Ki Han, June M. Kwak, Xingyun Qi

**Affiliations:** ^1^Department of New Biology, DGIST, Daegu, South Korea; ^2^Department of Biology, Rutgers University, Camden, NJ, United States

**Keywords:** stomatal lineage, development, transcription, environment

## Abstract

Stomata are micropores that allow plants to breathe and play a critical role in photosynthesis and nutrient uptake by regulating gas exchange and transpiration. Stomatal development, therefore, is optimized for survival and growth of the plant despite variable environmental conditions. Signaling cascades and transcriptional networks that determine the birth, proliferation, and differentiation of a stomate have been identified. These networks ensure proper stomatal patterning, density, and polarity. Environmental cues also influence stomatal development. In this review, we highlight recent findings regarding the developmental program governing cell fate and dynamics of stomatal lineage cells at the cell state- or single-cell level. We also overview the control of stomatal development by environmental cues as well as developmental plasticity associated with stomatal function and physiology. Recent advances in our understanding of stomatal development will provide a route to improving photosynthesis and water-stress resilience of crop plants in the climate change we currently face.

## Introduction

When plants transition from water to land, they became more exposed to carbon dioxide (CO_2_) and arid conditions. The evolution of stomata enabled plants to respond to the increased CO_2_ availability and limit water loss, ensuring their survival on land. Stomata are micropores on plant surface that are surrounded by two guard cells. These stomata open and close to facilitate gas exchange between plant inner tissues and the environment. Optimal gas exchange and water usage require efficient allocation of leaf surface to stomata. Improper stomatal patterning and density result in a change in mesophyll tissues and the epidermal area, affecting photosynthesis (Chen et al., 2016). Stomatal development, therefore, is tightly regulated to ensure there is no loss in photosynthesis efficiency.

**Figure 1:**
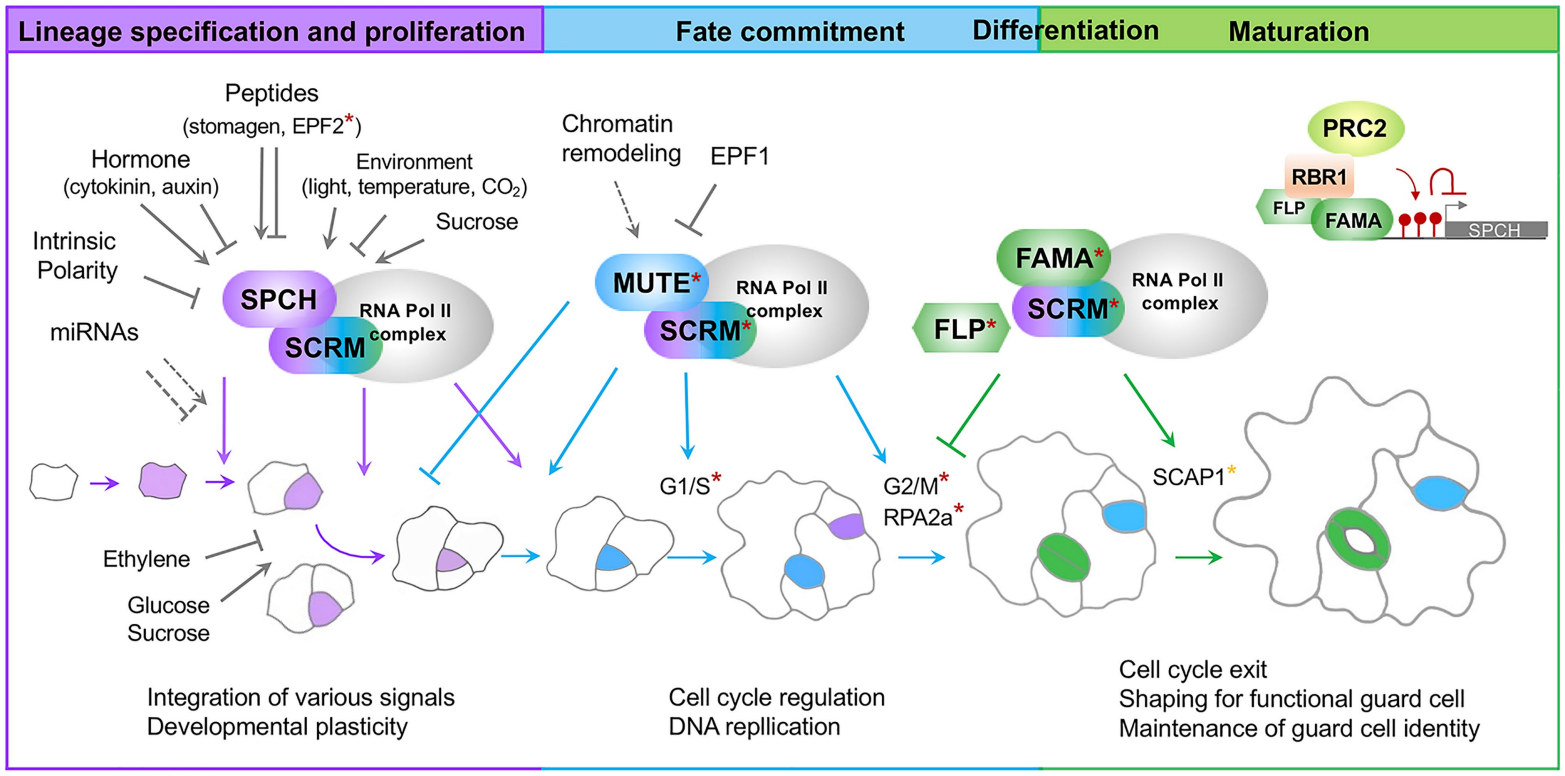
A current model for Stomatal development in Arabidopsis. Stomatal lineage progression is mediated by a sequential action of the developmental stage-specific transcription factors. SPCH/SCRM are responsible for lineage specification and proliferation; MUTE/SCRM for fate commitment; FAMA/SCRM for differentiation and maturation. Developmental and environmental signals primarily modulate the first step mediated by SPCH/SCRM. A subunit of the RNA polymerase II complex and associated proteins interact with core transcription factors. Direct targets for MUTE and FAMA are denoted by asterisks in red and orange, respectively. Cells highlighted in purple indicate stomatal lineage precursors. Cells highlighted in blue are late meristemoids and GMCs. Cells highlighted in green are immature and mature guard cells. Colored arrows indicate positive regulation of transcription factors. Colored, inverted Ts indicate negative regulation of transcription factors. Arrows and closed lines in gray indicate developmental and environmental factors regulating transcription factors or stomatal lineage cells. A dotted line indicates potential regulation. SPCH, SPEECHLESS; SCRM, ICE1/SCRM2; FLP, FOUR LIPS; RPA2A, a core subunit of Replication Protein A complexes; SCAP1, STOMATAL CARPENTER 1; RBR1, RETINOBLASTOMA-RELATED 1; PRC2, Polycomb Repressive Complex 2; EPF, Epidermal Patterning Factor; G1/S G2M, phases of cell cycle.

Stomata are found in most land plants (Duckett and Pressel, 2018), and the basic morphology is highly conserved (Chen et al., 2017). Most land plants, including Arabidopsis and grasses, undergo stomatal lineage progression and bear similar molecular components despite their substantial differences in morphology and arrangements (Raissig et al., 2016; Hepworth et al., 2018; Conklin et al., 2019; McKown and Bergmann, 2020). In dicotyledonous Arabidopsis, stomatal development initiates from a subset of protodermal cells, meristemoid mother cells (MMCs), which gain stem-cell fate (Figure 1). These MMCs undergo several rounds of amplifying division, where asymmetric division occurs in an inward spiral manner. Each round of asymmetric division yields a small daughter cell, a meristemoid, and a large daughter cell, a stomatal lineage ground cell (SLGC). Meristemoids become guard mother cells (GMCs) which further undergo a single round of symmetric division and differentiation. This process produces two mirror-symmetric guard cells with a pore in the center. SLGCs can continue with spacing division, in which they divide asymmetrically to generate satellite stomata, or they can differentiate into pavement cells (Han and Torii, 2016; Lee and Bergmann, 2019). The stomatal complexes of monocotyledonous grass species are distinct from those in eudicot. Dumbbell-shaped guard cells are accompanied by subsidiary cells, arranged in parallel to the leaf vein, and develop without stem cell-like meristemoid cells. The initiation of stomatal lineage by an asymmetric division of the precursor cells directly produces GMCs. The neighboring cells of the newly formed GMCs acquire subsidiary mother cell (SMC) fate and establish polarity toward the adjacent GMC. After the SMCs asymmetrically divide and differentiate into subsidiary cells, the GMCs divide symmetrically to generate a pair of guard cells. Two guard cells and subsidiary cells together form a dumbbell-shaped stomatal complex (Hepworth et al., 2018; Nunes et al., 2020).

Stomatal development is sequentially controlled by three basic helix–loop–helix (bHLH) transcription factors, SPEECHLESS (SPCH), MUTE, and FAMA. These transcription factors work together with their heterodimeric partners SCRM (ICE1) and SCRM2 (Ohashi-Ito and Bergmann, 2006; MacAlister et al., 2007; Pillitteri et al., 2007; Kanaoka et al., 2008; Figure 1). The SPCH/SCRM heterodimer determines stomatal fate and maintains the proliferation step by promoting asymmetric division (MacAlister et al., 2007). The MUTE/SCRM dimer terminates the proliferative state initiated by SPCH/SCRM and drives differentiation of meristemoids to GMCs (Pillitteri et al., 2007). The FAMA/SCRM dimer induces guard cell differentiation and restricts the last symmetric division to form a pair of guard cells (Ohashi-Ito and Bergmann, 2006). Two closely related R2R3 MYB transcription factor genes, *FOUR LIPS (FLP)* and *MYB88*, function in a semi-redundant manner with FAMA in differentiation and coordination of the terminal symmetric cell division (Geisler et al., 1998).

bHLH genes regulating stomatal development are well conserved across the land plants (Liu et al., 2009). However, their function is alternatively wired among different species to accommodate the fundamental difference in the patterning and shape of stomatal complexes. In particular, two copies of *SPCH* that function redundantly at the entry of stomatal lineage are found in Brachypodium and rice (Raissig et al., 2016). In contrast to Arabidopsis, MUTE protein in grass species can move from GMCs to the adjacent cells (Raissig et al., 2017; Wang et al., 2019; Wu et al., 2019). This lateral movement of MUTE establishes the identity of SMCs and drives the differentiation of subsidiary cells in a non-cell-autonomous fashion (Nunes et al., 2020).

Expression profiling of stomatal lineage cells at different developmental stages has revealed dynamic changes in the transcriptomes (Pillitteri et al., 2011; Adrian et al., 2015; Zhu et al., 2020; Ho et al., 2021). Moreover, direct target genes of the three master transcription factors have been identified (Hachez et al., 2011; Lau et al., 2014; Han et al., 2018). The recent advance in single-cell RNA-sequencing technology enables profiling spatiotemporal gene expression at the level of the individual stomatal lineage cells (Liu et al., 2020; Lopez-Anido et al., 2021). The systems-level analyses of stomatal lineage cells have deepened our understanding of how the developmental stage-specific transcriptional factors fulfill the developmental program.

Mitogen-Activated Protein Kinase (MAPK) cascades consist of YODA (YDA), MKK4/5/7/9, and MPK3/6. These cascades act upstream of the developmental stage-specific transcription factors (Bergmann et al., 2004; Wang et al., 2007). The activation of the MAPK cascade destabilizes SPCH and MUTE (Lampard et al., 2008; Qi et al., 2017). YDA-MPK3/6 MAPK signaling also plays a substantial role in establishing the cellular polarity required for asymmetric division and stomatal fate specification (Zhang et al., 2015, 2016). The molecular switch that either activates or suppresses the MAPK cascade, at least in part, consists of secreted epidermal patterning factor (EPF) peptides. EPF peptides are perceived by their corresponding cell-surface receptor complexes composed of the proteins belonging to the ERECTA family and their co-receptors TMM and SERK families (Torii, 2012). EPFs and their cognate receptors are conserved throughout the land plants (Caine et al., 2016; Hepworth et al., 2018). Functional studies of monocot species suggest that EPF1 and EPF2 inhibit, but EPFL9 enhances stomatal development, indicating that they are the functional ortholog of Arabidopsis EPFs (Franks et al., 2015; Hepworth et al., 2015; Wang et al., 2016; Hughes et al., 2017; Caine et al., 2019; Dunn et al., 2019; Mohammed et al., 2019). In addition, PANGLOSS1 and PANGLOSS 2 are Leucine-Rich-Repeat receptors in grasses and promote polarization of asymmetric SMC division (Cartwright et al., 2009; Facette and Smith, 2012; Facette et al., 2015). This result implies divergent receptor complexes perceive the extrinsic signal to modulate stomatal development.

Stomatal development is tightly controlled to ensure growth and survival and allows the plant to adapt to environmental changes. The current global climate changes could directly affect stomatal development and function and, thus, plant growth. CO_2_ is a gas that exacerbates the greenhouse effect and, therefore, global climate change (Lindsey, 2020). Temperature increases are often accompanied by drought stress due to enhanced water evaporation and changing rain patterns. Both excessive heat and drought stress negatively impact plant production. A significant loss (80–90%) in grain yield can result from warm temperatures during the plant reproductive stage (Hatfield and Prueger, 2015). Given the role of stomata in plant growth, further investigation of stomatal development and their response to environmental changes would provide a strategy that could enhance plant performance and productivity despite global climate change (Dow and Bergmann, 2014; Buckley et al., 2019).

## Transcriptional Control of Stomata Development

### Initiation and Maintenance of Stomatal Lineage Stem Cells

Stomatal lineage specification initiates with the *SPCH* expression in a subset of protodermal cells. This expression defines MMCs and continues through several rounds of asymmetric division. Persistent SPCH activity is found in meristemoids. The molecular signature of meristemoids was found *via* transcriptome analysis of stomatal development mutants overwhelmingly composed of meristemoids (Pillitteri et al., 2011). Pillitteri et al. identified the polarity protein POLAR and found that the cytokinin signaling pathway involving ARRs and CLEs, and ERECTA-family receptor-like kinases were part of the molecular feature of stem-cell populations.

How SPCH contributes to the specification and proliferation of stomatal lineage cells has been uncovered by identifying the targets of SPCH. Genome-wide profiling of SPCH targets was conducted by analyzing the transcriptome of transgenic plants harboring SPCH under the control of an inducible promoter and chromatin immunoprecipitation sequencing (Lau et al., 2014). The key regulatory genes, including *SCRMs, TMM, ERL2, EPF2, BASL, POLAR*, and the *ARK3/AtKINUa* kinesins, and brassinosteroid biosynthetic and response genes were found to be regulated by SPCH. The other genes identified as SPCH targets include *PHYTOCHROME-INTERACTING FACTOR 4 (PIF4;* Lau et al., 2018), *CLE9/10*, and *ARR16/17* (Vaten et al., 2018). These genes highlight the role of SPCH as an integrator conferring developmental flexibility of stomatal lineage in response to environmental or hormonal stimuli.

### New Players and Refining the Paradigm

A recent study using single-cell transcriptome analysis of stomatal lineage cells has proposed an extended role for SPCH in reinforcing cell fate decisions (Lopez-Anido et al., 2021). *SPCH* appears to be expressed beyond the early stages of stomatal development and co-expressed with either *MUTE* or *FAMA*. This observation was supported by a reporter gene analysis showing the *SPCH* expression in GMCs and a small population of young guard cells. These findings contradict the existing paradigm that stomatal development proceeds with the sequential actions of the master transcription factors in each state of transition. Furthermore, the coding sequence of *SPCH* did not fully complement the *spch* null mutants due to the disrupted level and timing of SPCH expression. This lack of complement resulted in a reduced number of stomata and defects in fate commitment to GMCs. Similar defects were observed in knock-down mutants for *SPCH* by artificial microRNAs (miRNAs) expressed under the *MUTE* promoter. Time-lapse imaging visualizing SPCH and MUTE protein expression showed overlapping expression patterns in late meristemoids (Davies and Bergmann, 2014). These studies indicate that SPCH activity is required for proper conversion of meristemoids to GMCs. It remains elusive, however, how SPCH functions in the later stages of stomatal development.

Another scRNA-seq analysis of stomatal lineage cells identified 11 cell clusters of epidermal cells, including two cell types that cannot be classified with known marker genes (Liu et al., 2020). One of the unclassified epidermal cell types highly expresses transcription factors and displays relatively similar patterns to early meristemoids in the developmental trajectory, suggesting that variable cell populations may exist at early stomatal developmental stages. This study also suggests potential regulators of stomatal development and a possible genetic network: *BASIC PENTACYSTEINE (BPC)* gene family and *WRKY33* genes are highly expressed in the MMC through GMC state. Higher-order BPC mutants exhibit defects in stomatal patterning and arrested precursors. Although SPCH was unaffected by the *bpc* sextuple mutants, the expression levels of SCRMs, MUTE, and FAMA were reduced. The detailed mechanism related to these transcription factors in stomatal development requires further investigation. Because the plant-specific BPCs family is involved in the recruitment of repressive histone-modifying complexes (Hecker et al., 2015; Mu et al., 2017; Xiao et al., 2017), it would be intriguing to test whether dynamic chromatin changes occur through the action of the BPC family during stomatal lineage progression.

### MUTE, a Potent Inducer of Cell Cycle Regulators for Stomata

MUTE terminates the self-renewing meristemoid state initiated by SPCH. It triggers unidirectional differentiation accompanied by a single symmetric division to create a stomate surrounded by a pair of guard cells (Pillitteri et al., 2007). Genome-wide transcriptome analysis of transgenic plants in which *MUTE* is chemically induced (*iMUTE*) revealed a comprehensive MUTE-dependent gene expression profile (Han et al., 2018). MUTE shares the targets of stomatal genes with SPCH for continued lineage progression. MUTE directly induces SCRMs, ERL1, POLAR, and BASL-like SPCH. In contrast, the earlier EPF2 signal induced by SPCH is attenuated by MUTE activation. The expression of the common receptors (*ERLs*), however, is maintained to receive the EPF1 signal. These findings suggest that MUTE serves as a transcriptional switch for proper stomatal patterning. The majority of GO categories of genes up-regulated in the iMUTE plants include cell cycle and cell division, which is unique to the *iMUTE* transcriptome compared to that of *iSPCH*. MUTE directly induces expression of cell cycle activators, CDKs and cyclins, followed by the activation of cell cycle repressors, FAMA and FLP, which in turn ensures symmetric division occurs only once (Han et al., 2018).

The scRNA-seq analysis further defines the molecular signature of fate commitment at single-cell resolution by showing how cell state-specific transcription factors, chromatin remodelers, and cell cycle regulators are dynamically regulated (Lopez-Anido et al., 2021). After the onset of fate commitment is triggered by MUTE, phase transition of the cell cycle is observed in discrete clusters. There, MUTE (G1/S of the cell cycle) and FAMA (G2/M of the cell cycle) are exclusively expressed.

The R2R3-MYB transcription factor FLP functions redundantly with FAMA for stomatal differentiation and direct repression of the cell cycle genes (Xie et al., 2010; Lee et al., 2014b). Enhanced terminal symmetric division in a loss-of-function mutant of FLP exhibits paired stomata. Yang et al. have shown that the paired stomata phenotype of *flp* mutants can be suppressed by introducing the *rpa2a* mutation. The RPA2a subunit of the Replication Protein A (RPA) complexes is responsible for DNA replication, recombination, and repair, functions cooperatively with CDKB1s and CYCA2s in restricting terminal symmetric division and in maintaining DNA content and guard cell size. Therefore, phenotypic suppression of the *flp* mutant by *rpa2a* mutation is likely due to the failure of cell cycle checkpoints without RAP2a (Yang et al., 2019). Triggered by CDKB1s, RPA2a phosphorylation is associated with nuclear localization and function in DNA replication processes. It appears that the expression of RPA2a and genes involved in DNA replication is increased in the *iMUTE* plants (Han et al., 2018).

These findings indicate that during stomatal formation, MUTE contributes to timely coordination of the cell cycle. Further investigation is required to unveil how the cell cycle and the chromatin landscape are linked to the termination of a self-renewing state and fate commitment.

### Fate Decision of Stomatal Lineage Ground Cells

SLGCs are large daughter cells derived from asymmetric divisions of MMCs or meristemoids. SLGCs bear bi-potency, meaning that they can either reenter asymmetric division to generate a satellite stomate or they can differentiate into a pavement cell.

Previously, it was shown that prolonged MUTE expression in 2-week-old *mute* mutants, where the meristemoids and SLGCs are arrested after several rounds of asymmetric division, resulted in clustered stomata (Trivino et al., 2013). In contrast, induced MUTE expression in wild-type plants did not show the clustered stomata phenotype due to full differentiation of stomatal lineage cells (Trivino et al., 2013). This result implies that SLGCs adjacent to the arrested meristemoid are competent to be a stomate. Additionally, it shows that SLGC differentiation to a pavement cell may require the MUTE activity in meristemoids.

Some molecular features of SLGCs were obtained from the transcriptome analyses of early stomatal lineage cells. It appears that SLGCs are enriched with genes associated with cell division and cytokinesis; they are poised between proliferation and endoreduplication-coupled differentiation (Ho et al., 2021). Fate decision to pavement cells or termination of proliferative SLGCs might also be linked to auxin-mediated onset of endoreduplication (John and Qi, 2008). It was reported that an auxin response gradient is formed and fluctuates within SLGCs, which helps to determine the fate and morphology of pavement cells (Grones et al., 2020). Cytokinin signaling, however, preferentially promotes asymmetric divisions in SLGCs (spacing division) through the induction of SPCH expression. SPCH directly regulates genes encoding the cytokinin inhibitory effector ARR16 and the secreted peptide CLE9. ARR16 reduces the cytokinin sensitivity of SLGCs, and CLE9 suppresses SLGC division. A negative feedback regulatory circuit, therefore, is formed between cytokinin and SPCH to fine-tune SLGC divisions (Vaten et al., 2018).

Despite the recent findings, the mechanism allowing SLGCs to maintain proliferate-state or to differentiate remains unknown. Although it is challenging to define the characteristics of SLGCs due to their heterogeneity, asynchronous production, and lack of specific SLGC markers, a detailed molecular characterization of SLGCs should enhance the investigation.

### Shaping Guard Cells and Maintenance of Guard Cell Fate

The final step of consecutive state transition in stomatal development is mediated by the transcription factor FAMA. It promotes guard cell differentiation and negatively controls symmetric division of GMCs (Ohashi-Ito and Bergmann., 2006). FAMA appears to be directly induced by MUTE. However, unlike other MUTE targets induced immediately, FAMA induction is delayed until just prior to symmetric division (Han et al., 2018). This delay implies there is an additional mechanism regulating FAMA expression. Hachez *et al* (Hachez et al., 2011) identified FAMA targets and regulators of guard cell development by transcript profiling of genes that are differentially modulated over chemically induced FAMA (*iFAMA*). These genes, regulated by FAMA, encode proteins with diverse functions, including transcription factors, cell cycle controllers, receptors, signaling proteins, and proteins associated with cell wall modification and metabolic processes (Hachez et al., 2011). This study supports the function of FAMA in differentiation of GMCs to guard cells and the maintenance of guard cell identity.

The loss-of-function mutant *stomatal carpenter 1* (*scap1*) develops skewed and dysfunctional guard cells. *SCAP1* encodes a Dof-type transcription factor and regulates the expression of key molecules in stomatal function and structure such as GORK (outward K^+^ channel), MYB60, and PME6 (pectin methyltransferase; Negi et al., 2013). As a consequence, *scap1* mutants display impaired ion homeostasis and esterification of extracellular pectins responsible for cell wall maturation lining the pores (Negi et al., 2013). The loss-of-function mutant for PME6 displays dysfunctional guard cell dynamics due to the mechanical change in the guard cells (Amsbury et al., 2016). The expression of *FAMA* and *SCAP1* in guard cells and the rapid up-regulation of *SCAP1* upon *FAMA* induction (*iFAMA*; Ohashi-Ito and Bergmann, 2006; Hachez et al., 2011; Negi et al., 2013) suggest that SCAP1 is a potential target of FAMA. Despite the role of SCAP1 during stomata maturation, the expression of *SCAP1* is observed in young leaf primordia. Loss-of-function mutants display a reduced stomatal density and patterning defects (Castorina et al., 2016). The change in stomatal density and increased patterning defects suggests that SCAP1 plays an additional role in stomatal development.

A component of the RNA polymerase II complex plays a role in stomatal differentiation in concert with FAMA (Chen et al., 2016). The hypomorphic mutant for NRPB3, the third largest subunit of the RNA polymerase II complex, produces more stomatal lineage-, paired-, and non-stomatal cells. Interestingly, the *nrpb3* mutant synergistically produces a tumor-like structure when combined with *fama* and *flp*. NRPB3 physically interacts with FAMA and SCRM, but not with SPCH and MUTE. RPAP2 IYO MATE (RIMA) is another protein interacting with NRPB3. RIMA also physically interacts with SPCH, MUTE, FAMA, and SCRM (Chen et al., 2021). It appears that the state-specific transcription factors associated with the general transcription machinery give rise to the cell state-specific regulation of gene expression. Stomatal lineage cell divisions are thereby limited at later stages of stomatal development.

Guard cells are the terminal state of stomatal cell lineage. Fully differentiated guard cells must irreversibly lose their proliferation ability to maintain cellular identity for stomatal function. Failure in maintenance reverts a guard cell to a stomatal lineage precursor, leading to a re-initiation of early stomatal lineage from a differentiated stomate. Previous studies have shown that the RETINOBLASTOMA RELATED (RBR)-FAMA and RBR-FLP interactions play a critical role in freezing the guard cell identity upon completion of differentiation (Lee et al., 2014a,b; Matos et al., 2014). These interactions mediate the RBR-mediated recruitment of Polycomb Repressive Complex 2 (PRC2) and establish the repressive histone modification H3K27me3 of the stomatal lineage genes (Lee et al., 2014a,b; Matos et al., 2014). This mechanism is partly supported by an analysis of the single-cell type transcriptome and histone modification (H3K27me3 and H3K4me3) dynamics in normal guard cells compared to the guard cells in the reprogramming state (FAMA^LGK^; Lee et al., 2019). When point mutations are introduced in the RBR binding motif (LxCxE) of FAMA (referred as FAMA^LGK^), FAMA^LGK^ is not capable of interacting with RBR and recruiting PRC2 (Matos et al., 2014). Modest changes in H3K27me3 peaks were observed between WT and FAMA^LGK^. In FAMA^LGK^ cells, the SPCH locus loses the H3K27me3 modification and its expression is concomitantly increased. The level of H3K27me3 at the MUTE and FAMA loci and their expression level, however, remained unchanged in the FAMA^LGK^ guard cells. This finding suggests that de-repression of SPCH may contribute to the re-initiation of the stomatal lineage in FAMA^LGK^ and that H3K27me3 is required to prevent guard cell fate from reverting to the precursor state. However, the MUTE expression level in FAMA^LGK^ is contradictory to the previous report where MUTE is highly up-regulated in 15-day-old cotyledons of FAMA^LGK^ (Matos et al., 2014).

SPCH expression alone is not capable of forming stomata (Davies and Bergmann, 2014). Ectopic SPCH expression results in more asymmetric divisions (Han et al., 2018). These results imply that the stomate in the stomate phenotype may not be solely attributable to the de-repression of SPCH. Knock-down of the PRC2 activity in guard cells mimics the stomate in the stomate phenotype with a very low frequency compared to FAMA^LGK^. This activity suggests a minor contribution of H3K27me3 and the presence of other mechanisms to maintain guard cell integrity. Indeed, H3K27me3 marks are increased on the locus of WOUND-INDUCED DEDIFFERENTIATION 1 (*WIND3*) during guard cell reprogramming (FAMA^LGK^), thereby resulting in transcriptional down-regulation of WIND3. The ectopic expression of *WIND3* using the SPCH promoter enhances the FAMA^LGK^ phenotype. These results imply that guard cell integrity is maintained by complex mechanisms in addition to the known H3K27me3 reorganization at the loci of stomatal lineage genes.

Using the proximity labeling method that identifies spatial and temporal information in protein–protein interactions, the nuclear proteome of young guard cells and novel FAMA-interacting proteins were identified (Mair et al., 2019). The FAMA interactors include bHLH transcriptional factors SCRM, transcriptional co-activators that link transcriptional factors to RNA polymerase II, and histone acetyltransferases, which is consistent with the previous study showing a link between FAMA and RNA polymerase II (Chen et al., 2016). FAMA also interacts with the transcriptional co-repressor TOPLESS-related proteins that recruit histone deacetylases to transcriptional factors and their linker protein that mediates the interaction between the co-repressor complex with transcription factors (Long et al., 2006). Among the highly abundant proteins in the nuclei of young guard cells, SHL (SHORT LIFE) binds to both H3K27me3 and H3K4me3. SHL functions as a histone reader to direct a particular transcriptional outcome. It might be involved in the establishment of repressive chromatins in concert with the co-repressors to lock the guard cell identity in their terminally differentiated state. Overall, FAMA could positively or negatively regulate target genes in concert with the co-activators and the co-repressors. This regulation is likely mediated through histone acetylation/deacetylation in response to developmental or hormonal signals.

## Signaling Cascade Converging to Transcription Factors

Plant survival dictates that the level of expression and the activity of the master transcription factors in each state of stomatal development must be tightly regulated. SPCH expression is modulated directly by upstream transcription factors. These factors are induced by light, drought, and heat (Klermund et al., 2016; Lau et al., 2018; Qi et al., 2019). SPCH activity is crucial to determine the fate of bi-potent stomatal lineage cells after asymmetric division and is substantially modulated by various kinases. MAPK signaling cascades inhibit the SPCH activity (Lampard et al., 2008; Gudesblat et al., 2012; Yang et al., 2015; Han et al., 2020). The mechanism by which the expression and activity MUTE and FAMA are regulated, in contrast, is not fully understood yet.

The signaling pathway upstream of the bHLH transcription factors is initiated by the EPF-family ligands, ERECTA-family receptor-like kinases, TMM as a co-receptor, and the downstream MAPK signaling cascade. The EPF ligands belong to the peptide family secreted to the apoplast (Herrmann and Torii, 2020). Structural and biochemical analysis revealed that heterodimerization between the ER-family receptors and TMM is required for EPF1 and EPF2 ligand perception, but was not required for EPFL4/5/6 (Lin et al., 2017), which indicates that ligand-based selectivity of receptor heterodimerization specifies the downstream biological process. ER receptor-TMM modules can also associate with the co-receptors BRI1-ASSOCIATED RECEPTOR KINASE/SOMATIC EMBRYOGENESIS RECEPTOR KINASE (BAK/SERK) to form a ternary receptor complex. This complex contributes to the stabilization of a specific ligand–receptor pair and hence, confers higher specificity in signaling (Meng and Yao, 2015).

EPF1 and EPF2 peptide ligands negatively regulate stomatal formation (Hara et al., 2007, 2009; Hunt and Gray, 2009). EPF2 restricts the initiation of stomatal formation by reducing the stability of SPCH (Bergmann et al., 2004; Pillitteri et al., 2011; Robinson et al., 2011). Exaggerated EPF2 signaling leads to a plant leaf epidermis composed of only pavement cells, similar to the *spch* mutants (Hara et al., 2007; Rowe and Bergmann, 2010; Rychel et al., 2010). *EPF2* expression is directly regulated by SPCH and SCRM (Lau et al., 2014; Horst et al., 2015). It is repressed by MUTE activity when the stomatal fate is committed in GMCs. This regulation allows EPF1 to access the common receptor system (Han et al., 2018). Another EPF-family member, EPF1, inhibits stomatal formation by negatively regulating the stability of the MUTE protein (Hara et al., 2007; Lee et al., 2012, 2015; Lin et al., 2017; Qi et al., 2017, 2020). Enhanced EPF1 signaling causes the stomatal lineage to arrest at the meristemoid state, phenocopying the *mute* mutants. Unlike *EPF2*, *EPF1* expression is not directly regulated by MUTE (Qi et al., 2017). The transcription factors regulating *EPF1* expression are unknown.

EPF-like 9 (EPFL9), also known as STOMAGEN, promotes stomatal formation and is highly responsive to environmental cues (Kondo et al., 2010; Sugano et al., 2010). With a highly similar topology structure, STOMAGEN competes with EPF1 and EPF2 for the same receptor complex but does not activate the MAPK cascade (Ohki et al., 2011; Lee et al., 2015; Qi et al., 2017). An auxin-related transcription factor MONOPTEROS (MP) suppresses the expression of *STOMAGEN*. The light-inducible transcription factor *ELONGATED HYPOCOTYL 5* (*HY5*) induces *STOMATAGEN* expression (Wang et al., 2021).

SCRM functions as a scaffold protein and associates with MPK3/6 and SPCH (Putarjunan et al., 2019). MPK3/6 first binds to the bipartite SCRM-KRAAM motifs of SCRM, which bridges with SPCH. Then, it triggers the subsequent phosphorylation of SPCH, resulting in the degradation of SPCH-SCRM (Putarjunan et al., 2019). The KRAAM motif is distinct from the putative MAPK docking sites and is uniquely found in SCRMs and their orthologs. This distinction suggests a mechanism for MAPKs conferring substrate specificity in a developmental process.

The localization of the MAPK cascade is unevenly distributed in MMC and in the two daughter cells derived from an asymmetric division. This distribution is facilitated by the intrinsic polarity protein, BASL. BASL arranges the tethering of YDA-MPK signaling components at its polarized crescent of the membrane, which results in elevated SPCH phosphorylation/destabilization to reduce the concentration of SPCH protein in SLGCs (Zhang et al., 2015, 2016). YDA-MAPK signaling must be restricted to allow high division potential in MMCs. This process is mediated by the polarized POLAR-BRASSINOSTEROID-INSENSITIVE2 (BIN2) module at the polarity crescent. Polarized BIN2 can inhibit YDA, leading to the attenuation of the YDA-MAPK signaling (Houbaert et al., 2018; Guo and Dong, 2019). The protein phosphatase BRI1 SUPPRESSPR 1 (BSU1)-LIKE 1 (BSL1) associates with the polarity complex and subsequently triggers the translocation of BIN2 from the polar crescent to the nucleus. It also activates YDA-MAPK signaling in SLGCs. BIN2 in the nucleus directly phosphorylates and destabilizes SPCH. This destabilization further restricts asymmetric division and leads to differentiation (Gudesblat et al., 2012; Guo et al., 2021a,b).

MAP KINASE PHOSPHATASE1 (MKP1) fine-tunes the MAPK signaling by inactivating the MAPK cascade at the early stage of stomatal development (Tamnanloo et al., 2018). The *mkp1* mutants undergo the asymmetric entry division but fail to differentiate, resembling the *mute* mutant phenotype. Genetic analysis puts MKP1 upstream of MPK3/6 and downstream of YDA. Additionally, MPK3/6 signaling is hyperactivated when MKP1 is absent, suggesting MKP1 deactivates MPK3/6. Interestingly, MKP1 expression fully rescues the *mkp1*mutant phenotype only when the MPK1 expression is driven by the SPCH promoter. This result suggests that MKP1 has a significant role in controlling guard cell fate commitment in early stomatal precursor cells.

Phosphorylation on the SPCH protein usually leads to its degradation. Yet, phosphorylation on Thr49, Thr50, Ser51, and Ser52 of the SPCH protein has been shown to stabilize SPCH (Han et al., 2020). The accumulation of KIN10, a catalytic subunit of the conserved energy sensor kinase SnRK1, can be induced by sucrose and promotes stomatal development. *KIN10* is highly expressed in the nuclei of stomatal lineage cells where it phosphorylates and stabilizes SPCH to promote sucrose-induced stomatal formation (Han et al., 2020). Mutations in *KIN10* result in decreased stomatal index, while overexpression of KIN10 results in a higher stomatal index. This finding partly explains the previously observed stomatal phenotype when plants are treated with high doses of sucrose (Akita et al., 2013).

Multiple kinases phosphorylate SPCH, resulting in the destabilization of SPCH. Protein phosphatases that are responsible for dephosphorylating SPCH, however, have recently been discovered (Bian et al., 2020). The scaffolding A subunit of the PP2A heterotrimeric complex directly associates with SPCH. Loss-of-function mutations in PP2A or inactivation by PP2A inhibitors results in reduced stomata formation and round pavement cells. This result suggests that PP2A promotes stomatal development by positively regulating SPCH stability. Furthermore, undifferentiated precursors after asymmetric divisions and round pavement cells in the *pp2a* mutants increase the likelihood that PP2A may regulate other proteins such as MUTE or those involved in pavement cell differentiation.

## Environmental Cues and Developmental Plasticity

**Figure 2:**
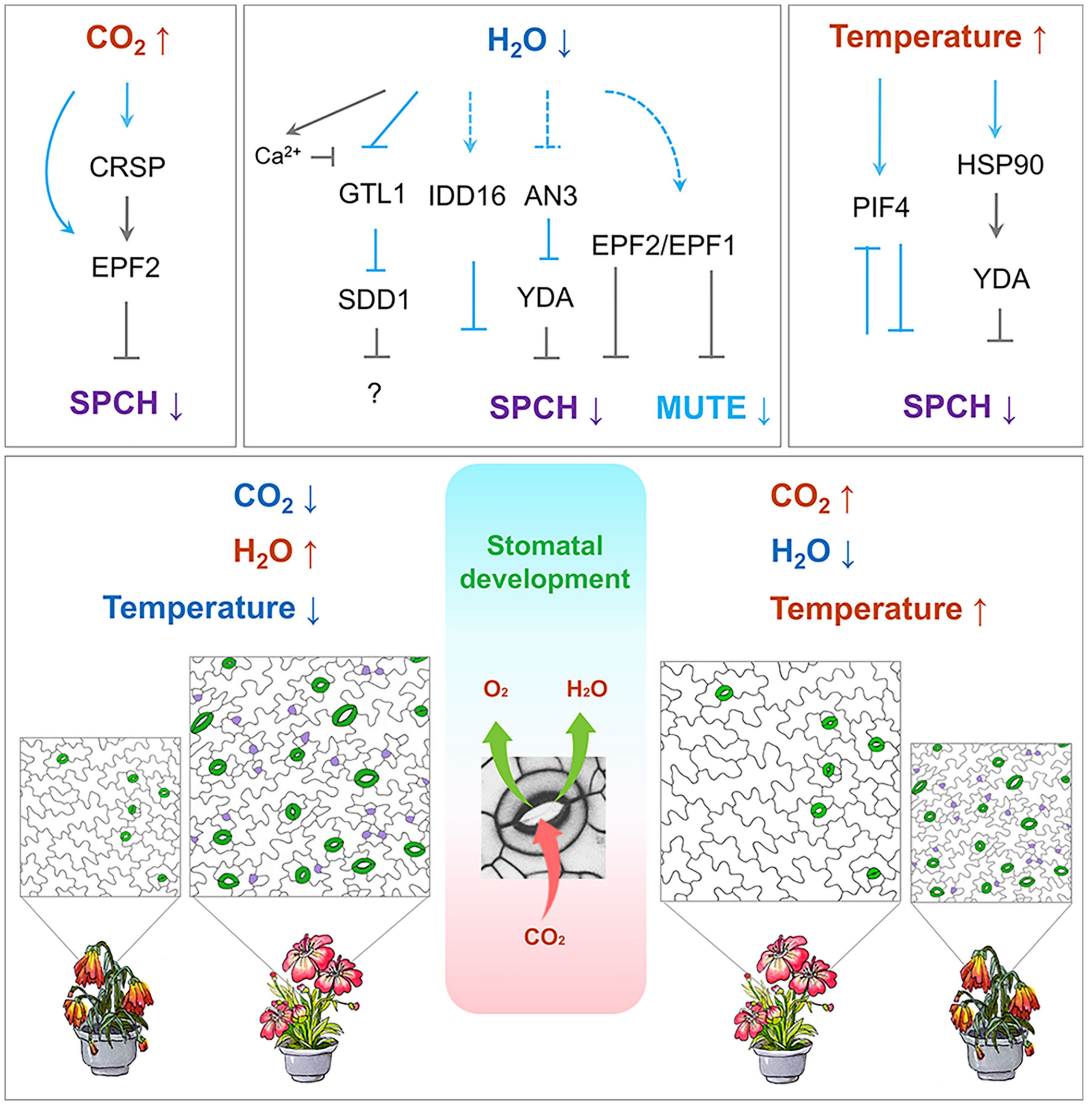
Regulation of stomatal development by environmental cues. **Top:** Temperature, CO2, and water availability modulate the signaling cascade, which leads to the regulation of SPCH and MUTE. Arrows indicate positive regulation, and inverted Ts indicate negative regulation. Solid lines in light blue indicate transcriptional regulation, and solid grey lines indicate post-translational regulation. Dashed lines indicate potential regulation. **Bottom:** A stomate, formed by a pair of guard cells, takes up CO_2_ (red arrow) for photosynthesis and releases the byproduct O_2_ (green arrow) and water vapor (green arrow). On the left: low CO_2_ concentration, sufficient water supply, and ambient temperature conditions prior to the global climate change. On the right: elevated CO_2_ concentration, deficient water supply, and increased atmospheric temperature conditions after global climate change. Stomata are highlighted in green, and meristemoids in purple. The flowering plants underneath each of the stomatal images represent the growth and stress response of the plants along with the stomatal density. Color code: blue indicates decrease; red indicates increase.

Given the essential role of stomata in photosynthesis and nutrient uptake, stomatal development is tightly controlled in response to a diverse range of environmental and phytohormonal signals (Qi and Torii, 2018). Plants have developed a highly coordinated regulatory mechanism that balances the need for photosynthesis and transpiration according to their specific environment (Hepworth et al., 2016). Atmospheric CO_2_ concentration, water availability, environmental temperature, light intensity, and nutrients all influence the endogenous program (Figure 2).

### Environmental Factors Affecting Stomata Development

#### Carbon Dioxide

Atmospheric CO_2_ significantly influences stomatal formation and function. Evolutionary studies over geologic time scale as well as ecological studies imply that atmospheric CO_2_ levels are inversely correlated with stomatal density and impacts on stomatal function (McElwain and Steinthorsdottir, 2017). Global climate impact assessments for crops have shown that elevated atmospheric CO_2_ levels may be a preferable condition to mitigate yield losses due to climate changes. This preference is because low atmospheric CO_2_ concentration imposes a negative repression on photosynthetic productivity (Sage and Coleman, 2001). In bread wheat, drought stress often causes reduction in seed yield, but this loss can be compensated by an elevated CO_2_ level. This compensation is likely due to the high CO_2_ concentration satisfying the photosynthesis needs more efficiently when stomatal conductance is limited by water shortage (Dunn et al., 2019). CO_2_ enrichment often comes with other climate changes including rising temperatures and water shortages. The response of decreasing stomatal density at a high CO_2_ level, therefore, implies that the ease in CO_2_ uptake allows plants to respond to other limiting factors such as water availability.

The current understanding of how CO_2_ regulates stomatal formation comes from a milestone study by Schroeder and his colleagues (Engineer et al., 2014; Figure 2). They found that *Arabidopsis* β-carbonic anhydrase double mutants (*ca1ca4*; Hu et al., 2010) display an increased stomata density at an elevated CO_2_ level. This increase in stomata density was due to compromised *EPF2* expression. This study identified CO_2_ RESPONSE SECRETED PROTEASE (CRSP), which is induced by elevated CO_2_ level and encodes a protease that specifically cleaves the precursor of EPF2 to release the mature peptide into the apoplast. The processed, functional EPF2 inhibits stomatal initiation by destabilizing SPCH. Mutations in either *CRSP* or *EPF2* impair the stomatal development influenced by elevated CO_2_ concentration (Engineer et al., 2014). Moreover, treatments with a high concentration of CO_2_ promote satellite stomatal formation, leading to changes in stomatal distribution on abaxial cotyledons (Haus et al., 2018; Higaki et al., 2020). A detailed molecular mechanism and the benefits for plants to have satellite stomata under high CO_2_ conditions are not fully understood.

#### Water Shortage

Water scarcity is a growing problem that correlates with global climate change. Drought conditions cause previously arable land to become unable to support crops. Increased drought conditions will cause crop yield reduction or even failure, which can result in starvation conditions for many populations. To avoid evaporative water loss, plants reduce stomatal conductance. This reduction in conductance ensures survival of the plant but causes a reduction in photosynthesis, yields, and productivity (Blum, 1996). Understanding how plants effectively use limited water resources is, therefore, crucial for future food security. Many studies on common crop plants indicate that reducing transpiration by decreasing stomatal density is a feasible way to increase plant drought tolerance without compromising yield (Franks et al., 2015; Hepworth et al., 2015; Wang et al., 2016; Hughes et al., 2017; Caine et al., 2019; Dunn et al., 2019; Mohammed et al., 2019).

Molecules upstream of the core pathway controlling stomatal development are the current targets for genetic manipulation. In poplar, dehydration or ABA treatment causes up-regulation of *PdEPF1* (Wang et al., 2016). Overexpression of *PdEPF1* consistently results in a low stomatal density, high water use efficiency, and drought tolerance (Wang et al., 2016). In creeping bentgrass, overexpression of Osa-miR393 up-regulated *EPF* expression. This up-regulation leads to reduced stomatal density and enhanced plant drought tolerance (Zhao et al., 2019). Similarly, overexpression of *EPF2* in Arabidopsis, rice, and barley causes reduced stomatal density, decreased transpiration rate, and improved water use efficiency (Franks et al., 2015; Hepworth et al., 2015; Hughes et al., 2017; Dunn et al., 2019) (Caine et al., 2019; Mohammed et al., 2019). Reducing stomatal density by regulating EPFs, therefore, can be a promising tool for breeding crops that can better withstand drier environments without significant yield loss.

STOMATAL DENSITY AND DISTRIBUTION 1 (SDD1) could be another molecular target for improving drought tolerance in plants. Knockout mutants for *SDD1* display a noticeably increased stomatal density with severe stomatal clustering (Berger and Altmann, 2000; Von Groll et al., 2002). Because SDD1 belongs to the subtilisin-like serine protease family, it has been proposed that SDD1 cleaves a precursor of a mobile peptide that negatively regulates stomatal development (Berger and Altmann, 2000; Von Groll et al., 2002). EPF1 and EPF2, however, work independently of SDD1 (Hara et al., 2007, 2009). Manipulation of SDD1 can optimize WUE and thus improve plant drought tolerance. In Arabidopsis, *SDD1* expression is significantly up-regulated upon drought (Yoo et al., 2019). Overexpression of wild tomato *SchSDD1* in Arabidopsis and tomato results in higher productivity under drought conditions (Morales-Navarro et al., 2018). Further, it was found that *SDD1* expression is regulated by GT-2 LIKE 1 (GTL1), which is a transcription factor that binds the GT3 box in the *SDD1* promoter to trans-repress its transcription (Yoo et al., 2010). GTL1 is downregulated by drought stress. In *gtl1* loss-of-function mutants, *SDD1* expression is significantly increased, causing a 25% reduction in stomatal density (Yoo et al., 2010). Consequently, WUE is higher and drought tolerance is enhanced (Yoo et al., 2010). The Ca^2+^-binding protein, calmodulin, upon binding of osmotic stress-induced Ca^2+^, interacts with one of the N-terminal helices of GTL1 and inhibits GTL1 binding to the *SDD1* promoter. This inhibition thereby de-represses the *SDD1* expression and improves WUE (Yoo et al., 2019).

GTL1 is also known to regulate trichome development. This regulation would imply a correlation between the two epidermal cell types. Indeed, WUE was higher in four tomato lines with higher trichome density. Trichome density negatively correlates with stomatal density, and the ratio of trichomes to stomata shows a strong positive correlation with WUE (Galdon-Armero et al., 2018). The same observation was also reported in a wild-type population of Arabidopsis (Simon et al., 2020). Not surprisingly, many molecules regulating trichome development also play a role in stomatal development (Torii, 2021).

Many environmental conditions have a significant effect on SPCH (Chen et al., 2020; Figures 1 and 2). Qi and colleagues found that plants with a lower concentration of SPCH protein exhibit significantly increases in drought tolerance (Qi et al., 2019). Overexpression of the C2H2 zinc-finger transcription factor INDETERMINATE DOMAIN 16 (IDD16) caused a reduction in the SPCH transcription in a dose-dependent manner. The ChIP analysis indicates that IDD16 directly associates with the SPCH promoter, suggesting that SPCH is a direct downstream target of IDD16. In line with this, the IDD16 RNAi plants exhibit a higher stomatal density. Plants overexpressing *IDD16* displayed a reduced stomatal density and were hypo-sensitive to drought stress (Qi et al., 2019).

ANGUSTIFOLIA3 (AN3) is another transcription regulator involved in drought tolerance and stomatal development (Meng and Yao, 2015). Without AN3, fewer stomata were produced, transpiration was reduced, and the plants displayed improved drought tolerance. In *an3* mutants, a drastic increase was observed in the *YDA* transcript level. The ChIP analysis corroborated the theory that AN3 binds to the YDA promoter, indicating that AN3 acts as a transcriptional repressor of *YDA*.

Overall, drought stress modulates the stomatal formation pathway almost at every step, including the peptide ligands, the MAPK cascade, and the downstream transcription factors. Interestingly, all these targets appear to be regulated at the transcriptional level, indicating that transcriptional regulation is an efficient, effective way to deal with environmental stress.

#### High Temperature

Responding to environmental temperature, plants adjust their cooling capacity by controlling the rate of transpiration. Besides the quick response of stomatal apertures, control of stomatal formation contributes to plant body temperature. For instance, under ambient conditions, well-watered plants with more stomata (such as *epf1epf2*) maintain low body temperature, whereas those with fewer stomata (such as EPF1 overexpression lines) show higher body temperature (Hepworth et al., 2015; Caine et al., 2019). When water is restricted, however, EPF1 overexpression lines display more stable and lower leaf temperature than lines with a higher stomatal density. These effects are particularly notable during the reproductive stage (Hepworth et al., 2015; Caine et al., 2019; Mohammed et al., 2019). More interestingly, when the environmental temperature rises, stomatal pores of the EPF1 overexpression plants were significantly larger than those of the wild-type plants. This change allowed the plants to compensate for lower stomatal density. Plants are, therefore, dynamically balanced between evaporative cooling and water conservation. They maintain this delicate balance by regulating stomatal conductance. Plants with fewer stomata display improved initial water conservation, which allows them to more efficiently regulate stomatal movements. This improved regulation ensures adequate evaporative cooling even under severe drought conditions at a high temperature.

When Arabidopsis plants grown at ambient temperature (22°C) are exposed to a high temperature (28°C), they develop fewer stomata (Crawford et al., 2012). PHYTOCHROME-INTERACTING FACTOR 4 (PIF4) is an essential component in this process. *PIF4* encodes a bHLH transcription factor involved in plant adaptation to high temperatures and is significantly up-regulated by elevated temperature (28°C; Koini et al., 2009). In stomatal lineage cells, high temperature stabilizes PIF4, which in turn binds to the *SPCH* promoter to repress its expression. This binding is responsible for inhibiting the entry of stomatal formation. Interestingly, *PIF4* was identified as an SPCH target, and the transcriptome data from the seedlings in which *SPCH* is conditionally expressed further indicate that SPCH protein also represses *PIF4* expression. Thus, at ambient temperature, SPCH promotes stomatal formation while blocking *PIF4* expression. When the temperature rises, *PIF4* is expressed and the stabilized PIF4 protein blocks *SPCH* transcription by binding to its promoter, therefore inhibiting stomatal formation through negative feedback (Lau et al., 2018).

HEAT SHOCK PROTEINS 90s (HSP90s) positively regulates YDA. Thus, YDA’s targets, MPK3 and MPK6, cannot be activated when HSP90s is depleted. Lack of activation results in insufficient SPCH phosphorylation and destabilization. A higher concentration of stable SPCH protein eventually leads to clustered stomata (Samakovli et al., 2020). HSP90s also affects YDA polarization *via* physical interaction in stomatal lineage cells. Moreover, loss-of-function mutants of YDA or HSP90s altered the transcript levels of at least 18 SPCH target genes. This alteration indicates that the YDA-HSP90s module negatively affects the transcriptional activity of SPCH (Samakovli et al., 2020).

Similar to drought stress, heat stress modulates the stomatal entry transcription factor SPCH and the MAPK cascade. Additionally, heat stress impacts protein stability and/or function.

#### Light

Light, essential for growth and development, acts as an energy source and a developmental signal in plants. Stomatal development is a trait of photomorphogenesis and is positively regulated by light (Casson et al., 2009; Kang et al., 2009). Plants detect light through photoreceptors, including phytochromes and cryptochromes (Paik and Huq, 2019). Mutations in these photoreceptors cause reduced stomatal density within the corresponding light spectra (Kang et al., 2009). A common target of light signals is CONSTITUTIVE PHOTOMORPHOGENIC 1 (COP1), which is an E3 ubiquitin ligase. When COP1 is destabilized by light, it no longer activates the downstream YDA cascade, which leads to stomatal formation (Kang et al., 2009). In dark conditions, active COP1 can degrade SCRM *via* its E3 ubiquitin ligase activity, thereby preventing stomatal development. This pathway occurs in a YDA-independent manner and does not affect SPCH stability, suggesting a parallel pathway of COP1 that modulates the abundance of SCRM under unfavorable light conditions (Lee et al., 2017).

Additionally, light promotes stomatal formation by inducing *STOMAGEN* (Hronkova et al., 2015). MONOPTEROS/ARF5, a member of the AUXIN RESPONSE FACTORs family, has been reported to associate with the *STOMAGEN* promoter to suppress its expression (Zhang et al., 2014). Whether light exposure regulates this auxin-related MONOPTEROS is unknown. A recent study found an integration point where light signals impinge on stomatal development (Wang et al., 2021). The light-responsive HY5 plays a role in fundamental developmental processes such as photosynthetic machinery assembly, chloroplast development, pigment accumulation, and photomorphogenesis (Gangappa and Botto, 2016). The HY5 genomic binding sites have revealed its hierarchical role in the light regulation of plant development (Lee et al., 2007). Wang and colleagues found that HY5, a central transcriptional factor, directly binds to the promoter and activates *STOMAGEN* expression in mesophyll cells (Wang et al., 2021). HY5 is required for light regulation of SPCH. In *hy5* mutants, SPCH is marginally maintained. Compromising HY5 regulation results in stomatal formation that is unresponsive to light intensity. Light-stimulated HY5 directly activates *STOMAGEN* expression in mesophyll cells, which in turn acts in the epidermis to stabilize SPCH. Knock-down or overexpression of *STOMAGEN* results in stomatal production insensitive to light intensity (Wang et al., 2021). The direct regulation of *STOMAGEN* expression by HY5 was also detected in a previous report by Zoulias *et al*. (Zoulias et al., 2020). The up-regulation of STOMAGEN by HY5 represents a regulatory mechanism of how an environmental signal is integrated into the developmental program. It provides a correlation between the CO_2_ uptake from stomata and the photosynthetic mesophyll cells.

#### Nutrients

Because nutrient uptake by roots is directly affected by transpiration, a correlation analysis of root and stomatal development in plants under drought conditions could be of interest. When plants are well watered, root area and the stomatal density are positively correlated. For example, *epf1epf2* mutants have an increased number of stomata and produce a larger root system than wild-type plants and thus, display improved phosphate and nitrogen accumulation (Hepworth et al., 2016). In contrast, plants with fewer stomata, such as transgenic plants overexpressing *EPF2*, share comparable root hair development and have a similar phosphate uptake capacity compared to wild-type plants (Hepworth et al., 2015, 2016). When water is restricted, a large decrease in stomatal density shows no significant effect on nitrogen accumulation (Hepworth et al., 2016). High transpiration rates due to increased stomatal density promote root development under water-sufficient conditions. When water shortage occurs, this correlation is altered, which implies that there is a complex interplay between root development and stomatal density.

miRNAs play a crucial role in plant development and environmental regulation (Song et al., 2019). It is surprising, then, that no miRNAs appear to target known stomatal regulators, especially given the considerable number of transcriptional factors and signaling proteins that modulate stomatal development. Loss-of-function mutations in *AGO1* (*ARGONAUTE 1*), however, drastically impact stomatal patterning and morphology of epidermal cells. This implies that miRNAs play some role in stomatal development (Yang et al., 2014). Recently, expression profiling of miRNAs in stomatal lineage cells was carried out using the cell state-specific promoters of *SPCH*, *EPF2*, *MUTE*, *EPF1*, and *FAMA* (Zhu et al., 2020). Most of the identified miRNAs appear to be expressed at the entry state of stomatal development and target genes involved in nutrient homeostasis, metabolism, and light response (Zhu et al., 2020). Up- or down-regulation of miRNAs results in defective stomatal patterning and density. Together with previous data, Zhu et al. suggest that stomatal lineage miRNAs and their dynamic changes constitute another layer of stomatal development regulation and may play a role in response to developmental factors, environmental cues, and nutritional status for proper lineage specification and/or progression at the entry of stomatal lineage.

Sugar status and ethylene signaling appear to fine-tune the intrinsic polarity and division potential of stomatal lineage precursor cells (Gong et al., 2021). CONSTITUTIVE TRIPLE RESPONSE (CTR1) is a core component that negatively regulates ethylene signaling. In the *crt1* mutants, the stomatal-specific polar protein BREVIS RADIX-LIKE 2 (BRX2) becomes depolarized, which reduces the amplifying asymmetric division. This phenotype can be alleviated by glucose or sucrose treatment, suggesting an antagonistic effect of ethylene and sugar signaling on the self-renewing capacity of stomatal linage stem cells. Because sugar is produced by photosynthesis occurring in mesophyll cells, the effect of nutritional status and environment on the overall leaf size and epidermal patterning suggests active communication between the epidermis and mesophyll cells.

## Conclusion and Perspectives

Significant progress has been made in stomatal development over the past two decades. The discovery of key regulators and a core developmental pathway in stomatal lineage has contributed to the current understanding of stomatal development. Technical advances have identified genome-wide transcriptomes at the bulk-tissue, single-cell types, or single-cell level. ChIP-sequencing data of the master transcription factors are also accumulating. Despite the recent discoveries, there are open questions to be addressed: How are the stomatal lineage cells first established? How is the expression of master transcription factors initiated and coordinated at the chromatin level? How is intrinsic polarity complex targeted to the cortical membrane and integrated into the extrinsic signaling pathway? What are the molecular links between the environmental and hormonal control of stomatal development? Given the wealth of genetic resources and various types of omics data, one should explore novel factors and investigate comprehensive gene networks in stomatal lineage.

Single-cell RNA-seq can be applied to profile responses to environmental or hormonal perturbations, which could reveal whether the cell identity and cells at different developmental stages differentially respond to stress or hormone. Profiling chromatin accessibility such as Assay for Transposase Accessible Chromatin Sequencing and deoxyribonuclease I hypersensitive sequencing at the single-cell level would further the current understanding of the transcriptional program when combined with the single-cell transcriptome. Additionally, advanced live-cell imaging and quantitative image analysis make it easier to keep track of lineage and monitor the cellular events at single-cell resolution, which will help to pave the way to applying the single-cell approach to crop species.

We are currently facing an environmental crisis as global climate change caused by the greenhouse gases accelerates the temperature rise and water shortage. Each of these factors will negatively impact plant ecosystems, sustainability, and food supply. Stomata can directly influence the atmospheric CO_2_ level, water evaporation, and even the surface temperature of the earth. Future challenges in stomatal development research, therefore, include addressing the open questions as well as translating discoveries made in Arabidopsis to agriculturally important crops. Many studies have demonstrated that stomatal morphology, density, size, distribution, and specialized cell type in grasses determine stomatal conductance. Stomatal development, then, significantly influences plant behavior in response to environmental changes. Genetic manipulation of stomatal development and physiology may contribute to better survival of plants and maintain agricultural productivity despite unfavorable conditions. Studies of stomatal development in monocots have started mostly by the comparative analysis of the genes in Arabidopsis. Because monocots bear linear arrangement of guard cells in specific cell files and four-celled stomatal complex, which enables “speedy stomata,” the investigation of stomatal development and their regulation is a key to the breeding guidance. As transcriptional regulation is the major mechanism in environmental regulation on stomatal development, the rapidly responding transcription factors could form a useful toolbox for genetic manipulation. The use of CRISPR/Cas9 genome-editing technology in a cell type-specific manner could overcome deleterious effects caused by the ubiquitous disruption of essential genes. It is necessary to collect useful stomatal traits for plants to better adapt to global climate changes, which will allow us to see the outcome of current efforts.

## Author Contributions

S-KH, JK, and XQ wrote the manuscript. All authors read and approved this version of the manuscript.

## Funding

The work in the authors’ laboratories was supported by grants from National Research Foundation (2019R1A2C3007376 and 2020R1A4A1019408). S-KH was supported by the Brin Pool Program funded by the Ministry of Science and ICT through the National Research Foundation (2020H1D3A2A02110999). XQ was supported by Rutgers University.

## Conflict of Interest

The authors declare that the research was conducted in the absence of any commercial or financial relationships that could be construed as a potential conflict of interest.

## Publisher’s Note

All claims expressed in this article are solely those of the authors and do not necessarily represent those of their affiliated organizations, or those of the publisher, the editors and the reviewers. Any product that may be evaluated in this article, or claim that may be made by its manufacturer, is not guaranteed or endorsed by the publisher.
